# A bi-directional neuro-robotic system to study computational properties of cell assemblies

**DOI:** 10.1186/1471-2202-12-S1-P37

**Published:** 2011-07-18

**Authors:** Jacopo Tessadori, Marcello Mulas, Paolo Massobrio, Sergio Martinoia, Michela Chiappalone

**Affiliations:** 1 Department of Neuroscience and Brain Technologies, Italian Institute of Technology, Genova, 16163, Italy; 2 Department of Biophysical and Electronic Engineering, University of Genova, Genova, 16145, Italy

## 

Behaviors, from simple to most complex, require a two-way interaction with the environment and the contribution of different brain areas depending on the orchestrated activation of neuronal assemblies. Understanding how information is coded and how synaptic mechanisms are implemented in physiological as well as pathological networks is one of the major challenges of neuroscience.

Researchers have begun to study the mechanisms of adaptive behaviors interfacing biological models of the brain with robots by means of electrophysiological techniques [1]. Among the others, dissociated cultures coupled to MEAs (i.e. Micro Electrode Arrays) provide a suitable experimental substrate for embodied electrophysiology since they allow long-term multisite recording and stimulation [2,3]. Here we will present what the main components of an experimental system are, in order to support the execution of neuro-robotic experiments. To establish a bidirectional communication between the neuronal preparation (network module) and a mobile robot (robotic module), the electrophysiological signals need to be translated into motor commands for the robot (decoding of neural activity), and at the same time the sensory signal from the robot need to be translated into a pattern of electrical stimulation (coding of sensory information). Both modules can be either real or simulated. More specifically: (i) the network module can be a culture of dissociated cortical neurons coupled to a commercial Micro Electrode Array (MCS, Reutlingen, Germany) or a simulation of that obtained by our simulator NeuVision, which allows to simulate large scale neuronal networks [4]; (ii) the robot can be the commercial mobile robot Khepera, manufactured by K-Team (Yverdon-les-bains, Switzerland) or its simulation, again obtained by NeuVision.

We fully simulated a closed-loop experiment in which a robot, controlled by the electrical activity of a neural network, moves in a circular arena containing some obstacles. To characterize the propagation of either spontaneous or evoked network bursts (i.e. a pattern of activity which involve almost the entire neuronal network), we made use of the center of activity trajectory (CAT) [5]. We observed that CATs of evoked network bursts follow typical paths, while CATs of spontaneous network bursts are not regular. Moreover we fully simulated a closed-loop experiment where the CAT method was used to generate motor commands. The obtained control of the robot allowed to accomplish an obstacle avoidance task.

Real modules have also been assembled and tested: preliminary experiments have been performed implementing a simple decoding strategy based on the mean firing rate of a culture region. Similarly, robot sensors provided information on obstacles distance which has been coded with a frequency-modulated electrical stimulation.

In conclusion, we think that a synergistic approach taking advantage of both simulated and real components could be crucial to increase the effectiveness of electrophysiological experiments in order to understand how networks encode information. The modularity of the software architecture makes easy to interface within the same experiment either real or simulated components such as large-scale neural network and robots.
